# Protein kinase C is involved with upstream signaling of methyl farnesoate for photoperiod-dependent sex determination in the water flea *Daphnia pulex*

**DOI:** 10.1242/bio.021857

**Published:** 2016-12-13

**Authors:** Kenji Toyota, Tomomi Sato, Norihisa Tatarazako, Taisen Iguchi

**Affiliations:** 1 Okazaki Institute for Integrative Bioscience, National Institute for Basic Biology, National Institutes of Natural Sciences, Department of Basic Biology, Faculty of Life Science, SOKENDAI (Graduate University for Advanced Studies), 5-1 Higashiyama, Myodaiji, Okazaki, Aichi 444-8787, Japan; 2 Environmental Genomics Group, School of Biosciences, University of Birmingham, Edgbaston, Birmingham B15 2TT, UK; 3 Graduate School of Nanobioscience, Yokohama City University, 22-2 Seto, Kanazawa-ku, Yokohama 236-0027, Japan; 4 Ecotoxicity Reference Laboratory, Risk Assessment Science Collaboration Office, Center for Health and Environmental Risk Research, National Institute for Environmental Studies, 16-2 Onogawa, Tsukuba, Ibaraki 305-8506, Japan

**Keywords:** *Daphnia pulex*, Environmental sex determination, Juvenile hormone, Methyl farnesoate, Photoperiod, Protein kinase C

## Abstract

Sex determination of *Daphnia pulex* is decided by environmental conditions. We established a suitable experimental system for this study using *D. pulex* WTN6 strain, in which the sex of the offspring can be controlled by photoperiod. Long-day conditions induced females and short-day conditions induced males. Using this system, we previously found that methy farnesoate (MF), which is a putative innate juvenile hormone molecule in daphnids, is necessary for male sex determination and that protein kinase C (PKC) is a candidate factor of male sex determiner. In this study, we demonstrated that a PKC inhibitor [bisindolylmaleimide IV (BIM)] application strongly suppressed male offspring induction in the short-day condition. Moreover, co-treatment of BIM with MF revealed that PKC signaling acts upstream of MF signaling for male sex determination. This is the first experimental evidence that PKC is involved in the male sex determination process associated with methyl farnesoate signaling in daphnid species.

## Abstract (precis)

**Summary:** This study is the first experimental evidence that PKC is involved in the male sex determination process associated with methyl farnesoate signaling in daphnid species.

## INTRODUCTION

The micro-crustacean water flea, genus *Daphnia*, is a representative zooplankton in freshwater inland ecosystems. They have a unique sex determination system, which is tightly correlated with changeable habitat conditions. Under favorable environmental conditions, mothers exclusively produce female offspring via parthenogenesis, whereas under an unfavorable environment, such as low temperature and short-day length, they begin to produce parthenogenetic male offspring instead (Banta and Brown, 1929; Hobæk and Larsson, 1990; Kleiven et al., 1992).

Previous studies have demonstrated that juvenile hormone (JH) or its mimics could induce male offspring production in daphnid species even under female-producing conditions (Olmstead and LeBlanc, 2002; Tatarazako et al., 2003). Therefore, JH is considered to be a male sex determinant. Additionally, the JH-sensitive period for male offspring production occurs during late period of oocyte maturation, prior to embryo release into the brood pouch (Ignace et al., 2011; Kato et al., 2011). However, the regulatory mechanisms of a JH surge during this critical interval have not been elucidated yet due to the lack of a male-inducible system without JH treatment.

To fill this knowledge gap, we have recently established a female- or male-inducible system using *D. pulex* WTN6 strain. The sex ratio of a clutch of this strain can be easily controlled by changing the photoperiod; a mother produces female progeny under long-day conditions (14 h light:10 h dark), whereas male progeny exclusively emerge under the short-day conditions (10 h light:14 h dark) (Toyota et al., 2015a). Based upon this induction system, we demonstrated that methyl farnesoate (MF) is likely an innate JH in daphnids (Toyota et al., 2015a), and furthermore, *N*-methyl-D-aspartate (NMDA) receptors (a subtype of ionotropic glutamate receptors) act as upstream regulators of MF signaling during male offspring production (Toyota et al., 2015b). In addition to NMDA receptors, expression levels of serine/threonine kinase-coding genes were varied in both up- and downstream of MF signaling (Toyota et al., 2015b). We hypothesize that protein kinase C (PKC) might be a primary candidate factor of MF signaling since some previous works indicated that JH signaling is transduced via PKC activation (Yamamoto et al., 1988, 1997). However, whether PKC is involved in the MF pathway triggering male sex determination in daphnids is still largely unknown.

In this study, we conducted a pharmacological assay of PKC inhibitor treatment of the WTN6 strain, and demonstrated that this treatment strongly suppressed male offspring induction even under male-inducible (short-day) conditions. This is the first experimental evidence that PKC is involved in male sex determination associated with MF signaling, and provides a new picture of the signaling network underlying environmental sex determination in *Daphnia*.

## RESULTS AND DISCUSSION

**Fig. 1.**
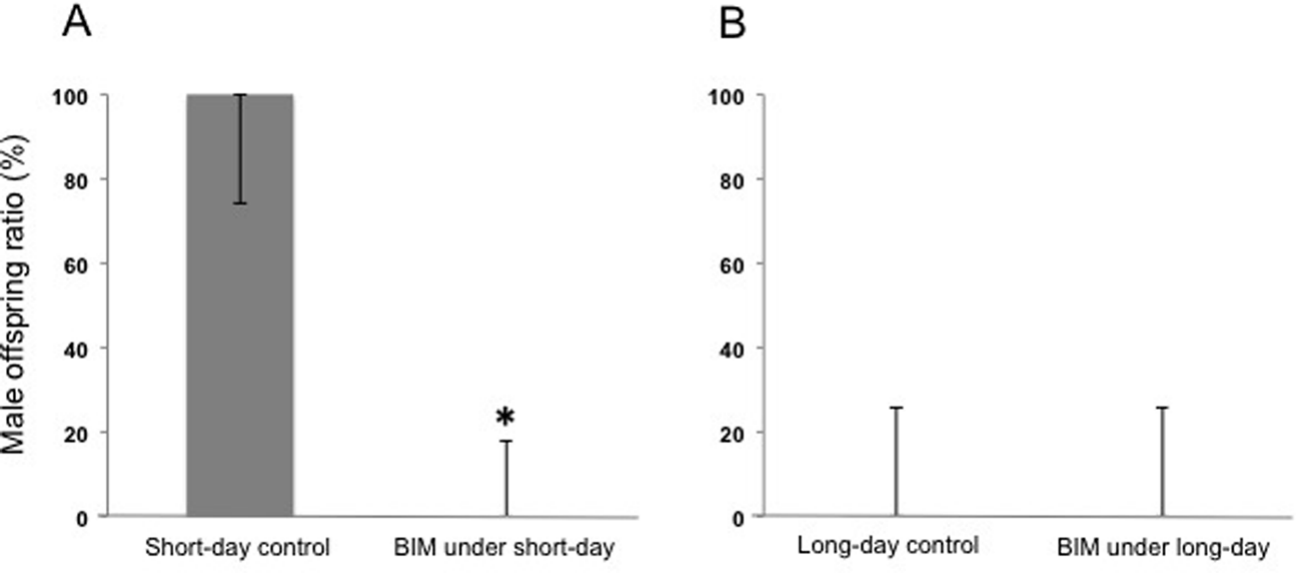
**Effect of PKC inhibitor, BIM, on the inducibility of male offspring.** (A) and (B) show data of short-day and long-day conditions, respectively (*n*=10-15). Y-axis values indicate the percentage of male offspring in the brood. Asterisk indicates significant difference compared to the control (Fisher's exact probability test, *P*<0.01). BIM was administered at 10 µM in short-day and long-day conditions, with DMSO as control solvent. Bars indicate the 95 % confidence interval.

To investigate the involvement of PKC in male offspring production under short-day conditions in *D. pulex*, we compared the sex ratio of offspring produced by females under PKC inhibition to control conditions for both long-day and short-day environments. Bisindolylmaleimide IV (BIM) was used as a PKC inhibitor (Denning et al., 1998). In the short-day control group (DMSO-treated), all mothers produced 100% male offspring, whereas in short-day BIM-treated groups, all mothers produced only female-offspring (Fig. 1A). In contrast, administration of BIM did not affect the proportion of female-producing mothers reared under the long-day condition (Fig. 1B). These results strongly suggested that PKC is involved with the male-sex determining process in WTN6 strain.

**Fig. 2.**
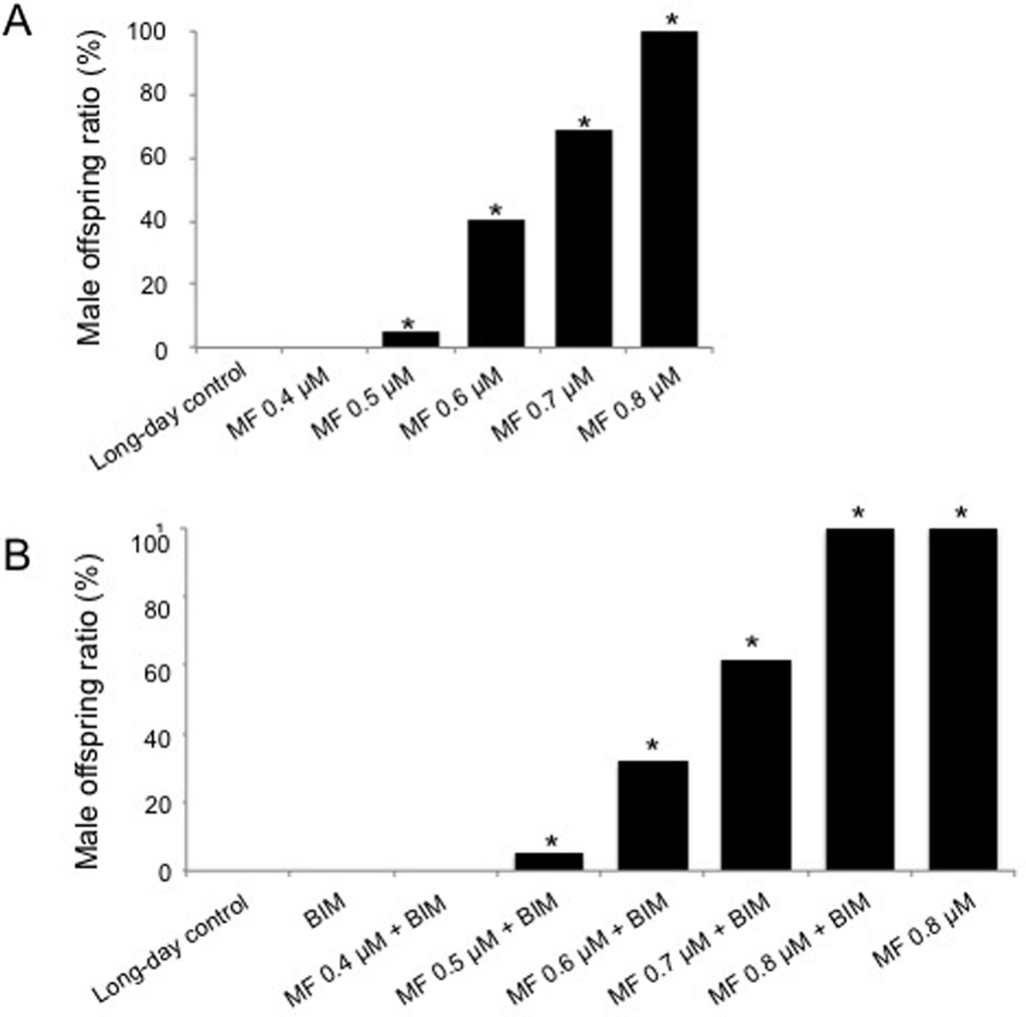
**Dose-dependent effects of methyl farnesoate (MF) with BIM on the inducibility of male offspring under long-day condition.** (A) and (B) show data of MF and MF with BIM, respectively (*n*=16 and 15). Y-axis values indicate the percentage of male offspring in the brood. Asterisks indicate significant differences compared to respective controls (Fisher's exact probability test, *P*<0.01). Concentration of BIM was 10 µM.

Next, we conducted the co-treatment of BIM with several concentrations of MF for dose-dependent male-inducible rates to clarify the hierarchical relationship between these signaling pathways. As shown in Fig. 2A, male offspring ratio showed an apparent increase in a dose-dependent manner to MF treatment. As a result of the co-treatment experiment, male induction rate did not change (Fig. 2B), indicating that PKC acts upstream of the MF signaling required for male sex determination.

Recent efforts have revealed that Methoprene-tolerant (MET) acts as a JH intracellular receptor in insects (Ashok et al., 1998; Jindra et al., 2013). In the presence of JH, MET binds to the partner protein SRC, which is also known as TAI in *Drosophila melanogaster* and FISC in *Aedes aegypti*, and forms the MET-SRC complex to activate its downstream factors (Charles et al., 2011; Li et al., 2011; Zhang et al., 2011). Moreover, it has been demonstrated that two daphnid species, *D. pulex* and *D. magna*, have an intracellular JH reception mechanism using MET-SRC complex that is similar to insects (Miyakawa et al., 2013). In addition to the intracellular JH signaling process, several lines of evidence have suggested that some actions of JH occur through membrane receptors via PKC signaling in male accessory glands in *D. melanogaster* (Yamamoto et al., 1988) and in ovarian follicles in *Locusta migratoria* (Zhou et al., 2002). Additionally, it has been reported using *Drosophila* cell line that PKC mediates phosphorylation of JH receptors to modulate binding of its receptors to JH-responsive elements (Kethidi et al., 2006). Furthermore, JH signaling via the PKC pathway may be widely present among crustaceans. For example, MF induces larval metamorphosis via PKC activation in the barnacle *Balanus amphitrite* (Yamamoto et al., 1997). Although PKC is considered as a crucial player in downstream mediation of JH signaling for non-genomic response, we reveal here that PKC also acts upstream of MF synthesis in daphnids.

**Fig. 3.**
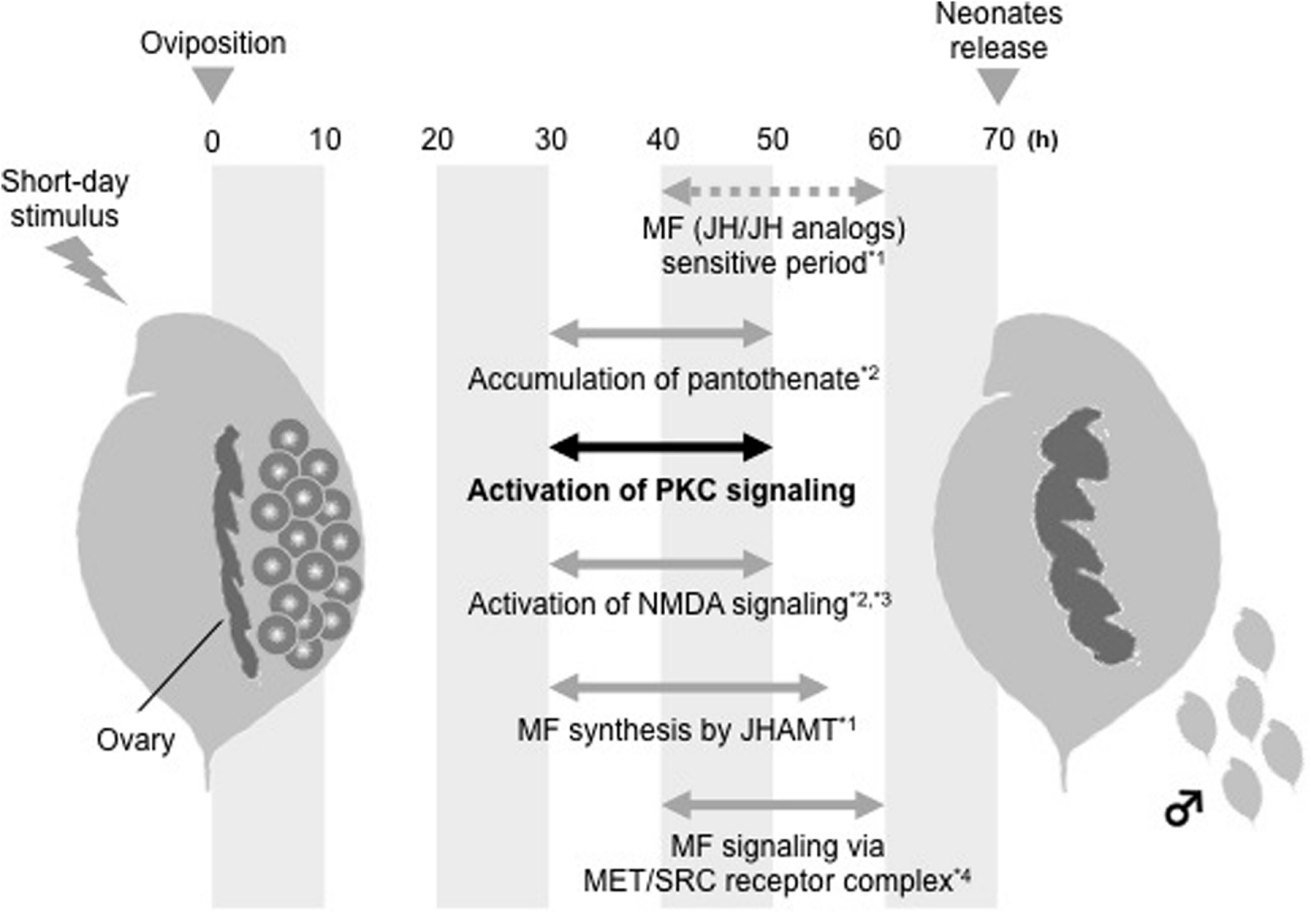
**Schematic illustration of a putative male sex-determining process triggered by short-day stimulus in *D. pulex* WTN6 strain.** MF (JH/JH analog)-sensitive period corresponds to an oocyte maturation period (40-60 h after oviposition) inside of mother's ovary. *1: Toyota et al. (2015a); *2: Toyota et al. (2015b); *3: Toyota et al. (2016); *4: Miyakawa et al. (2013).

Furthermore, our previous transcriptome analysis revealed that NMDA receptors act as an upstream factor of MF signaling (Toyota et al., 2015b); therefore, the next question addresses the regulatory relationship between the NMDA receptor and PKC involved with the MF synthesis in WTN6. Although these relationships have not been clarified yet in arthropods, including insects and crustaceans, a previous report has shown that PKC increases the channel­-opening rate of NMDA receptors and induces a rapid delivery of functional NMDA receptors to the cell surface in *Xenopus* oocytes expressing NMDA receptors (Lan et al., 2001). This knowledge provides an important hint in the interpretation of our results and enables us to build the following hypothesis about male sex determination in *Daphnia* species: when a mother receives the short-day stimulus, (1) PKC is activated and recruits NMDA receptors, (2) NMDA receptors mediate MF synthesis via expression of juvenile hormone *O*-methyltransferase (JHAMT) (Toyota et al., 2015a, b), and (3) MF binds to MET-SRC receptor complex to activate its downstream cascades for male sex determination (Miyakawa et al., 2013) (Fig. 3). To test this hypothesis and investigate whether this mechanism can be applied for other daphnid species, further experiments such as treatments of PKC activators will be necessary.

In this study, we demonstrated that PKC is involved with the male sex determination process in *D. pulex*. Moreover, co-treatment of PKC inhibitor with MF revealed that PKC acts upstream of MF signaling. This is the first evidence that PKC might be involved in MF synthesis. Although more detailed investigations examining the role of PKC in male sex determination process in daphnids will be required, our current findings provide important clues and a new hypothesis of the signaling network underlying photoperiod-dependent sex determination process in *D. pulex*.

## MATERIALS AND METHODS

### *Daphnia pulex* strain and its female- or male-producing conditions

The WTN6 strain of *D. pulex,* obtained from the Center for Genomics and Bioinformatics (Indiana University, IN, USA), was maintained at the National Institute for Basic Biology (Aichi, Japan) for more than 2 years. This strain was reared in dechlorinated fresh water, which was aerated and filtered through activated carbon for 2 weeks prior to use. A 0.04-ml suspension of 4.3×10^8^ cells ml^−1^ of *Chlorella vulgaris* was added daily to each culture (40 individuals/2 l). In this strain, the production of female and male offspring can be induced by rearing under 14 h light:10 h dark (long day) or 10 h light:14 h dark (short day) conditions, respectively (Toyota et al., 2015a). We determined the sex of offspring based on the length of the first antenna (Tatarazako et al., 2003).

### Chemicals and treatment procedure

We used bisindolylmaleimide IV (BIM; ≥98%; Sigma-Aldrich, St. Louis, MO, USA) as an inhibitor of PKC (Davis et al., 1992), and methyl farnesoate (MF; Echelon Bioscience, Salt Lake City, UT, USA) as a JH. Dimethylsulfoxide (DMSO; Nacalai Tesque, Kyoto, Japan) and dimethylformamide (DMF; analytical grade, Wako, Osaka, Japan) were used as solvent controls for BIM and MF, respectively, at concentrations below 0.01% (v/v). BIM dissolved in DMSO and MF dissolved in DMF were stored as 15 mM and 4 mM stock solutions, respectively, and kept at −20°C until use. All experiments (experiments 1 and 2) were conducted in 5 ml media in a 5 ml sampling tube (INA OPTICA, Osaka, Japan) containing one adult female (one-month-old or older) 30 h after ovulation, which is just before the MF-sensitive period. A total of 10-16 individuals were used for each treatment as replicates. In experiment 1, females were transferred to one of two ambient conditions: short-day or long-day, with a subset of females in each also exposed to BIM (10 µM). In experiment 2, all females were subjected to the long-day conditions. Animals were exposed to several concentrations of MF (0.4, 0.5, 0.6, 0.7 and 0.8 µM) either with or without a dose of BIM (10 µM). We tried the BIM exposure experiments using 15, 20, and 25 µM, however, all test animals died within 48 h after exposure. We checked sexes of all offspring produced and difference of sex ratio between treatments were statistically analyzed by Fisher's exact probability test with Holm's correction using R 2.15.3 (R Development Core Team, 2013).
